# Multi-level influences on increased overdose risk behaviors during the COVID-19 pandemic among people who use drugs in Rhode Island: a qualitative investigation

**DOI:** 10.1186/s12954-023-00741-w

**Published:** 2023-02-04

**Authors:** Lisa Frueh, Alexandra B. Collins, Roxxanne Newman, Nancy P. Barnett, Josiah D. Rich, Melissa A. Clark, Brandon D. L. Marshall, Alexandria Macmadu

**Affiliations:** 1 Department of Environmental and Occupational Health, Drexel University Dornsife School of Public Health, 3215 Market Street, Philadelphia, PA 19104 USA; 2 Department of Epidemiology, Brown University School of Public Health, 121 South Main Street, Box G-S-121-2, Providence, RI 02912 USA; 3 Department of Behavioral and Social Sciences, Brown University School of Public Health, 121 South Main Street, Providence, RI USA; 4 The Center for Health + Justice Transformation, The Miriam Hospital, 1125 North Main Street, Providence, RI USA; 5 Department of Health Services, Policy & Practice, Brown University School of Public Health, 121 South Main Street, Providence, RI USA

**Keywords:** Overdose risk behaviors, Drug use, Pandemic, COVID-19

## Abstract

**Background:**

The ongoing COVID-19 pandemic has disproportionately affected structurally vulnerable populations including people who use drugs (PWUD). Increased overdose risk behaviors among PWUD during the pandemic have been documented, with research underscoring the role of influencing factors such as isolation and job loss in these behaviors. Here, we use qualitative methods to examine the impact of the COVID-19 pandemic and pandemic-related response measures on drug use behaviors in a sample of PWUD in Rhode Island. Using a social-ecological framework, we highlight the nested, interactive levels of the pandemic’s influence on increased overdose risk behaviors.

**Methods:**

From July to October 2021, semi-structured interviews were conducted with 18 PWUD who self-reported any increase in behaviors associated with overdose risk (e.g., increased use, change in drug type and/or more solitary drug use) relative to before the pandemic. Thematic analysis was conducted using a codebook with salient themes identified from interview guides and those that emerged through close reading of transcribed interviews. Guided by a social-ecological framework, themes were grouped into individual, network, institutional, and policy-level influences of the pandemic on drug use behaviors.

**Results:**

Individual-level influences on increased overdose risk behaviors included self-reported anxiety and depression, isolation and loneliness, and boredom. Network-level influences included changes in local drug supply and changes in social network composition specific to housing. At the institutional level, drug use patterns were influenced by reduced access to harm reduction or treatment services. At the policy level, increased overdose risk behaviors were related to financial changes, job loss, and business closures. All participants identified factors influencing overdose risk behaviors that corresponded to several nested social-ecological levels.

**Conclusions:**

Participants identified multi-level influences of the COVID-19 pandemic and pandemic-related response measures on their drug use behavior patterns and overdose risk. These findings suggest that effective harm reduction during large-scale crises, such as the COVID-19 pandemic, must address several levels of influence concurrently.

## Introduction

The ongoing COVID-19 pandemic has amplified drug use and exacerbated ongoing epidemics of nonfatal and fatal overdose globally [1], and this effect has been particularly outsized in the USA. During the pandemic, self-reported substance use has increased dramatically across the USA [2–6], as have rates of nonfatal and fatal overdose [7, 8].

Among people who use drugs (PWUD), COVID-related response measures such as shelter-in-place policies altered the drug use risk landscape and culminated in heightened overdose risk [9, 10]. Several investigations in the USA, the UK, and Canada have also documented an increase in behaviors associated with overdose risk among PWUD during the pandemic. Reported overdose risk behaviors included increased drug use amount or frequency, using alone more often, and change in drug type due to pandemic-related supply changes [11–19]. Further, some studies have documented reduced access to harm reduction and treatment services among PWUD due to pandemic-related closures, further exacerbating overdose risk [11, 13, 16, 20]. The extant literature on the experiences of PWUD during the COVID-19 pandemic has demonstrated complex and multifactorial relationships between pandemic-related stressors and behaviors associated with increased overdose risk. However, few studies to date have leveraged social-ecological models to interrogate the interconnected, multi-level influences of the COVID-19 pandemic on overdose risk behaviors.

Bronfenbrenner’s social-ecological model provides a framework for understanding health that emphasizes nested levels of influences [21]. Applying the social-ecological model to health behavior, McLeroy et al. articulated five levels of influence specific to health behavior, which were adapted to guide this analysis: intrapersonal (individual) factors, interpersonal processes and primary groups (network factors), institutional factors, community factors, and public policy [22]. In the context of PWUD, examples of factors influencing drug use behaviors at each level include: drug use history, attitudes, and knowledge (individual level); friends and/or roommates’ drug use behaviors (interpersonal/network level); availability of treatment, recovery, and/or harm reduction services and programs (institutional level); community norms around drug use and inter-organizational cooperation across services and programs (community level); and local drug policy, ordinances affecting service availability, and Good Samaritan laws (public policy level). Importantly, social-ecological models posit that the multiple levels of influence are interactive, suggesting the utility of multi-level interventions for health promotion [23, 24].

Characterizing the multi-level influences of the pandemic on any increase in overdose risk behaviors among people who use drugs is particularly important within the Rhode Island context, where overdose deaths increased by more than 28% from 2019 to 2020 [25]. During emergent crises such as the COVID-19 pandemic, social-ecological models can provide systematic frameworks to guide time-sensitive research and intervention among people who use drugs during the pandemic [26]. Further, qualitative methods prove particularly salient in guiding time-sensitive research and intervention, as these approaches reflect ground truth for those most impacted. Here, we seek to explore and describe the nested and interactive influencing factors on drug use behaviors during the ongoing COVID-19 pandemic.

## Methods

### Recruitment and eligibility

Participants enrolled in the Rhode Island Prescription and Illicit Drug Study (RAPIDS) cohort were invited to participate in this qualitative investigation between July and October 2021. RAPIDS is an ongoing randomized clinical trial testing the efficacy of a fentanyl overdose education and test strip distribution intervention among a prospective cohort of PWUD in Rhode Island, and the trial has been described in detail elsewhere [27]. In brief, RAPIDS study participants are recruited using field-based recruitment strategies (e.g., targeted canvasing, word of mouth), web-based advertising (e.g., Craigslist), and advertisements on public transportation statewide (e.g., advertisements on bus routes that operate throughout the state) [27]. RAPIDS participants were recruited into this qualitative study via informational flyers provided by research assistants at baseline and follow-up survey visits. Prospective participants were referred to the senior author (AM) for further information about the study and eligibility screening, as described previously [28].

Eligibility criteria for this qualitative study were the same as RAPIDS and included: being 18–65 years of age, having Rhode Island residency, being able to complete interviews in English, providing informed consent, and self-reporting use of heroin, illicit stimulants, counterfeit prescription pills, or any drug by injection in the previous month. Additionally, eligible participants were required to have known someone who experienced an overdose in the 90 days prior to their interview [28]. Women (including transwomen) were purposively sampled to ensure gender-specific experiences were adequately captured. In sum, 25 RAPIDS participants were recruited for semi-structured interviews, which were performed between July and October 2021.

Given that the focus of the present study is on increased overdose risk behaviors during the COVID-19 pandemic (here, defined as March 2020 onward), participants who reported either increased protective behaviors (e.g., decreased drug use amount/frequency) or no change in overdose risk behaviors during the pandemic (*n* = 7) were excluded from the qualitative analyses described below, leaving a final analytic sample of 18 participants.

### Semi-structured interviews

In-person, semi-structured interviews were conducted by AM. Prior to conducting interviews, participants provided written informed consent. Interviews were facilitated using an interview guide that included topics focused on the pandemic’s impact on drug use and overdose risk behaviors (e.g., type of drugs used, amount and frequency, drug use partners and location, change in drug use behaviors during the pandemic). Interviews averaged 35 min, were audio recorded, and professionally transcribed with identifying information removed to protect confidentiality. Participants were compensated $30 USD cash for their time and were provided a packet of mental health and harm reduction resources following their interview. While no participants exhibited imminent risk of harm, protocols were established to discontinue study procedures and contact the study’s licensed medical provider if needed. This study was approved by the Brown University Institutional Review Board.

### Sociodemographic information and drug use patterns

Sociodemographic information and drug use patterns were collected using an interviewer-administered questionnaire. The following were sociodemographic characteristics were assessed: participants’ age, sex assigned at birth (male; female), current gender identity (male; female; transgender; genderqueer or nonbinary; and other), race (American Indian or Alaska Native; Asian; Black, African, Haitian, or Cape Verdean; Native Hawaiian or other Pacific Islander; white; biracial or multiracial/mixed race; and other), Hispanic or Latinx ethnicity (yes vs. no), and sexual orientation (straight; gay or lesbian; bisexual; queer; and other). Drug use patterns including prior week and lifetime drug use (heroin, fentanyl, prescription opioids, prescription stimulants, powder cocaine, benzodiazepines, crystal methamphetamine, club drugs, psychedelics, marijuana) and prior year experience with overdose (any vs. none) were also assessed.

### Thematic analysis

Analysis of the de-identified transcripts was performed using Dedoose 9.0.46 [29]. Using a thematic analysis approach, the lead author developed a codebook derived from interview guides (deductive codes) and emergent themes from repeated active reading of transcripts (inductive codes) informed by the following nested levels per the social-ecological model: individual, network, institutional, and public policy [21, 30–32]. Following McLeroy’s [22] social-ecological model for health promotion programs, the community level is defined as involving community-level norms as well as relationships between the organizations represented at the institutional level. Themes corresponding to this level did not emerge from interviews with this sample of participants; thus, our analysis utilizes the four remaining levels of McLeroy’s social-ecological model. During analysis, it was determined that inductive thematic saturation was achieved [33]. That is, through active reading of the transcripts, inductive codes were added until no new codes emerged through repeated readings of the included transcripts. Two members of the study team (LF and AM) discussed and refined the codebook before the lead author applied the final codebook to all de-identified transcripts. Pseudonyms were assigned to all participants using an online random name generator.

### Reflexivity statement

Interviews were conducted in a private room in a university building by a member of the study team (a Black/biracial cisgender woman) with extensive experience working with PWUD in the research setting. Transcripts were analyzed by a member of the study team (a white nonbinary person) who did not participate in any of the interviews or interact directly with participants. All co-authors have experience working with PWUD in research and/or outreach settings, and one or more co-authors have lived experience with drug use.

## Results

The median age of the 18 participants included in this analysis was 38 (IQR 32–49), and the majority were male (56%), white (78%), not Hispanic or Latinx (94%), straight (83%), and cisgender (100%). The most commonly reported drugs used in the week prior to interview were crack cocaine (89%), fentanyl (67%), and heroin (61%) and a majority (72%) of the participants reported experiencing at least one overdose in the prior year. Additional sociodemographics and drug use patterns of participants are presented in Table 1. As noted above, we included only participants who reported any increase in overdose risk behaviors during the pandemic in the following analysis. Excluded participants (who reported no change or decreased overdose risk behaviors, *N* = 7) did not differ meaningfully from included participants with respect to sociodemographic characteristics or type of drugs used. Notably, excluded participants were less likely to have experienced an overdose in the prior 12 months compared to included participants (14% vs. 72%).

**Table 1:** Sociodemographics and drug use patterns of participants

Characteristic	*N* = 18
*Sociodemographics*	
Age (Median [IQR]	38 [32, 49]
Sex at birth	
Male	10 (56%)
Female	8 (44%)
Cisgender	17 (100%)
Race	
Black^a^	2 (11%)
White	14 (78%)
Biracial or multiracial	2 (11%)
Ethnicity (Hispanic or Latinx)	1 (6%)
Sexual orientation	
Straight	15 (83%)
Bisexual	3 (17%)
*Any drug use: prior week*	
Heroin	11 (61%)
Fentanyl	12 (67%)
Prescription opioids	7 (39%)
Prescription stimulants	1 (6%)
Powder cocaine	9 (50%)
Crack cocaine	16 (89%)
Benzodiazepines	8 (44%)
Crystal methamphetamine	4 (22%)
Club drugs (e.g., Ecstasy)	2 (11%)
Psychedelics (e.g., acid)	0 (0%)
Marijuana	10 (56%)
*Any drug use: lifetime*	
Heroin	17 (94%)
Fentanyl	16 (89%)
Prescription opioids	18 (100%)
Prescription stimulants	13 (72%)
Powder cocaine	18 (100%)
Crack cocaine	18 (100%)
Benzodiazepines	16 (89%)
Crystal methamphetamine	12 (67%)
Club drugs (e.g., Ecstasy)	15 (83%)
Psychedelics (e.g., acid)	11 (61%)
Marijuana	18 (100%)
*Overdose experience*	
Overdose experience, prior 12 months^b^	13 (72%)

^a^Includes self-identified African, Haitian, and Cape Verdean ancestry

^b^Refers to having personally overdosed

Participants reported an increase in several behaviors associated with overdose risk, including increased drug use, an increase in solitary use, and/or changes in drug type during the pandemic. The most cited factors influencing the aforementioned drug use patterns were boredom, changes in the local drug supply, reduced access to services, and job loss and/or business closures. In the following sections, we explore participants’ overdose risk behaviors in the context of participants’ described individual-, network-, institutional-, and public policy-level drivers of increased overdose risk behaviors during the pandemic.

### Individual level

#### Anxiety, depression, and loneliness

Several participants cited exacerbated depression or anxiety as a reason for increased overdose risk behaviors during the pandemic. ‘Keith,’ a 31-year-old white man, indicated that anxiety about news related to the pandemic exacerbated his drug use, and that the isolation associated with social distancing led him to use alone more often:

*I feel like I’m using more 'cause I mean, my anxiety’s through the roof. We don’t know what’s gonna happen. You know what I mean? Talking about a second wave wiping out a ton of people and there’s a new strain that’s even more [...] And I don’t know. I mean this vaccine, you know, who knows? I’m thinking the consequences are gonna be disastrous. […]* [COVID] *like kind of distanced people. You know what I’m saying? So like it’s more like using alone now than it was before the pandemic.*

‘Devon,’ a 38-year-old white Hispanic man, described how the pandemic interacted with his existing depression:

*Well, it’s *[the pandemic’s] *made me more isolated, so that alone affects the depression and feeds it, and then the drugs feed into it. So it's just been a monster. It’s like a monkey on your shoulder, I guess. You know, it’s, uh, it's had a very negative impact as far as that.*

Similarly, ‘Kieran,’ a 34-year-old white man, noted that “*when everything was closed and people, like, they're just isolated at home, I mean-I know, uh, I called my-my person [seller] couple more times than usual*.”

‘David,’ a 51-year-old white man, expressed how his homelessness exacerbated feelings of isolation: “[During lockdowns,] *can’t see nobody, can't be around nobody. Everybody’s gotta be separated in the beginning and everybody was scared and I was homeless, so it’s tough*.”

‘Anthony,’ a 29-year-old white man, explained that he was using alone in his car more frequently than before the pandemic due to others’ unwillingness to host guests in their homes:

*I can’t go inside a lot of people’s houses now, you know? They’re so worried. So I end up just having to use in my car, which sucks 'cause it’s very risky using in your car. 'Cause, you don’t know what’s gonna happen, you know? You never know who might roll-up. Cops might roll… You know what I mean?*

For ‘Damaris,’ a 50-year-old Black woman, increased drug use was one way she coped with stress induced by pandemic-related financial strain:

[My drug use is] *getting worse 'cause the landlord and everybody stressing me out. I can’t do none of my appointments 'cause I don’t got money. I don’t got rent checks, and I’m just going nuts. I don’t know what to do.*

For many participants, uncertainty about the COVID-19 pandemic intersected with their mental health in ways that caused them to increase drug use to mitigate exacerbated feelings of anxiety, depression, and stress. Further, social distancing and lockdowns led to more frequent solitary drug use and/or increased drug use overall to manage fear, anxiety, depression, and loneliness. Though these excerpts underscore how individual-level factors such as stress and anxiety precipitated increased substance use, these emotional states were also connected to factors at other more distal levels of the social-ecological model, including the institutional (e.g., missed appointments) and public policy (e.g., social distancing) levels.

#### Boredom

Several of participants attributed their increased drug use to boredom. For example, ‘Kendrick,’ a 21-year-old multiracial man noted, “[the pandemic] *made me use more because of boredom*.” Like ‘Kendrick,’ ‘Anthony,’ a 29-year-old white man, further explained how the pandemic created feelings of boredom which impacted his drug use practices:

*It's like, now there's completely nothing to do, you know? And one of my main things for drug use is boredom. Boredom, you know? Like, I’m bored. So, I might as well just do some drugs […] I’m my own worst enemy. But, COVID didn’t help at all. 'Cause like now, there’s nothing to do. […] It’s opening back up and everything now, you know what I mean? But I’m saying before, you know, there was nothing… You can’t do anything. So what else is there to do? Let’s just do some drugs […] There’s nothing to do. I do drugs and work on my car, you know? And that’s literally my life right there.*

Similarly, ‘Lola,’ a 44-year-old Black woman, linked boredom to increased Percocet use:

*There was, like, totally nothing to do. I would have to go pick up my prescription and I feel during the pandemic is when I started taking them more. Because, at first, I was just taking ‘em when I was supposed to. Like, if I felt pain…but then, during the pandemic, you’re sitting there and you’re like, shit. I don’t have nothing to do. What are you gonna do? I could call the bootlegger. Stock up on liquor and then oh, wait. I’ve got my pills.*

Participants also noted feelings of collective boredom that encouraged more use among their peers:

*With COVID, for a while, everybody didn’t know what to do. So people are just around, walking around, like what are you doing? Like nothing. What are you doing? Nothing. Let’s do* [some drugs], *you know*? (‘Devon’)

‘Alisa,’ a 51-year-old white woman, shared a similar sentiment, linking a disconnection from social networks with boredom:

*It was like nobody was having parties […] there was really nothing going on. Some people sit home and eat and watch TV, you know, other people sit home and do drugs. And I would do like all three. […] Sit at home, eat, do drugs, you know? That’s how* [the pandemic] *affected me, I probably did* [drugs] *more*.

Boredom was the most common individual-level influence to which participants attributed changes in drug behaviors during the pandemic. For most participants, lack of daily structure during the pandemic due to lockdowns and social distancing led to increased drug use as a substitute activity. These excerpts again highlight the nested interactivity of individual-level factors within contextual influences on overdose risk behaviors.

### Network level

#### Supply/access changes

Many participants reported that changes in the local drug supply affected access to their preferred substances. This led participants to turn to other, less familiar drugs to replace those they had difficulty accessing. Many participants described an increased presence of fentanyl in the drug supply during the pandemic.

##### Crack cocaine

Participants explained that crack cocaine was more difficult to access during the pandemic and that fentanyl was commonly mixed into the crack cocaine that they were able to access locally. ‘Carlo,’ a 37-year-old multiracial man noted that “*you aren’t able to get it *[crack] *as easy*,” while ‘Shelly,’ a 47-year-old white woman explained that “*they’re mixing fentanyl with absolutely everything out there*.” For some participants, changes in the crack cocaine supply led to the use of alternative replacement substances. For others, they described paying more for crack cocaine or having to expand the network of sellers that they engaged with to buy crack cocaine. ‘Shelly’ expressed that:

*At the beginning *[of the pandemic],* there was a shortage on *[crack] *cocaine […] they almost doubled it in price. So it was hard to get in the very beginning. But I have, like, a lot of resource. I have like 13 crack dealers.*

Similarly, ‘Keith’ shared that there was a “*cocaine drought ‘cause the pandemic. And everything shut down and this and that, so things weren’t really coming in, and prices went through the roof*.” He speculated further that pandemic lockdowns contributed to supply shortages:

*Well, I mean, obviously if the country’s shut down—I’m talking about from a logistical standpoint. You know what I mean? And, like I said, I was in prison and I heard about, ‘Oh there’s no coke on the street,’ ‘the prices went through the roof…’ This and that because there’s no boats going out and planes and whatever. You know what I’m saying?*

##### Benzodiazepines

Other participants shared that benzodiazepines were more difficult to find during the pandemic, leading some to replace benzodiazepines with alternative substances, such as crack cocaine. For example, ‘Colton,’ a 24-year-old white man, shared that people were less willing to meet to sell benzodiazepines during the pandemic:

*A lot of people are unwilling to meet to sell them* [benzodiazepines], *or they’re selling them wholesale to other people because they’re… Kinda like how people were stocking up on toilet paper for a while there, basically. It became much more difficult to acquire, I would say. Specifically for benzos. But also for fentanyl to an extent, I would say […] It was worse before, but it’s getting better. Normally, um, especially with benzodiazepines, you could walk past a crowd and just, you know, ‘Hey, who has benzos,’ and you would be able to find them. Someone would be willing to sell them. They wouldn’t be suspicious of you.*

‘Devon’ reported similar difficulty accessing street-purchased benzodiazepines (benzos) during the pandemic, which led him to begin using crack cocaine:

*Normally, my favorite thing is pills. I’m a pill person. That’s how I originally got into the drugs, which is common now, you know. […] Doing crack, maybe it would come up once every three or four months. Now it’s the crack every day for whatever reason. 'Cause it’s there. It’s easier to get. Benzos now are very hard to get because they’re cracking down on it.*

##### General supply changes

Contrary to the reticence of sellers described by some participants, others suggested that sellers may feel more comfortable with the anonymity of wearing face masks during the pandemic, leading to greater willingness to sell. ‘Kieran,’ a 34-year-old white man explained:

*I think more people or the dealers out there might be pushin’ a lot more, mainly because of, like, the masks thing and things are shut down.* [Sellers] *can move around a little bit more when people are more isolated in their home […] Everyone wearin’ masks, and everyone drivin’ around with masks, and like, no one can pick out or, “Oh, I know that person. He’s a dealer,” […] I just think that they've been out there more than usual 'cause they’re able to cover their face up a little bit.*

Though many participants reported changes in the local drug supply, specific changes varied between participants. Crack cocaine shortages and price changes were reported by some, while others noted that crack had proliferated in the drug market. Similarly, while some participants indicated an abundance of fentanyl in the local drug supply, a few noted difficulties acquiring fentanyl. The effect of drug supply changes during the pandemic was fairly consistent across participants, with most who indicated drug market changes also indicating that they substituted their preferred substance with alternative substances due to these changes.

#### Housing-related social network changes

Several participants noted that a change in living situation and related social network composition changes during the pandemic influenced the amount or type of drugs they use. ‘Jessika,’ a 33-year-old white woman, shared that because of pandemic-related job disruptions:

*We couldn’t afford our apartment. So, we moved to this really cheap one […] And a lot of people there use. And my use has increased and the conditions that everything… Like, we used to just use heroin. Then we moved here. And now the people there smoke* [crack] *a lot.*

Other participants did not directly attribute housing changes during the pandemic to pandemic-related factors. Nevertheless, social network changes associated with changing living situations affected the drug use behavior of several other participants. For ‘Robert,’ a 34-year-old white man, his living situation during the pandemic intersected with a lack of daily structure, leading to increased drug use:

*Well, I mean,* [during the pandemic] *everything else drops out of the foreground and you live in a shitty transitional housing building. And the guy across the hall from me has dope every time. You have 15 dollars together, it becomes a problem very, very quickly when you’re not doing anything else.*

Similarly, ‘Alisa,’ a 51-year-old white woman explained why she began using crack cocaine more often:

*I think it’s the people I live around now. A lot of people do that, and I hate it. I hate that they all use that 'cause it’s always around and someone’s always like knocking on the door, ‘Hey you wanna get…’, and it’s kinda hard to say no when you’re an addict.*

‘Kieran,’ a 34-year-old white man, noted that a change in housing-related factors altered his drug use behaviors during the pandemic:

[Crack cocaine use] *was kinda impacted when I moved to that room, like, eight months ago and just gettin’ to know a couple of the guys that already lived there and what they do when they know-found out what I do. […] I think it was just the environment that I’d put myself in.*

Several participants shared that their housing situation changed during the pandemic, which caused changes in social network composition that led to increased use or a change in drug type—both behaviors associated with increased overdose risk.

### Institutional level

#### Reduced access to services

Some participants also described difficulty accessing harm reduction supplies, such as sterile needles. ‘David’ and ‘Keith’ explained that pandemic lockdowns made finding harm reduction supplies difficult. ‘David’ noted that everything “*being closed and not being able to find the place to go get stuff* [harm reduction supplies] *made it difficult for everything*.” Similarly, ‘Keith’ said that he had difficulty accessing supplies “*when everything was shut down*.”

Two participants shared that while street outreach provided harm reduction supplies during lockdowns, accessing these infrequent resources was difficult. ‘Anthony,’ a 29-year-old white man, explained:

*I haven’t been able to go to the needle exchange…I don’t know what time they’re open and what not. I mean, the dude comes down to Kennedy Plaza sometimes. But I always miss him for some reason. I always seem to miss him […] And I can't get no needles.*

Sharing similar frustration with spotty street outreach efforts, ‘Colton’ suggested that pharmacies should provide harm reduction supplies to increase access:

*Occasionally, there would be people walking around passing out naloxone […] But I think pharmacies should also be giving away certain things for free, which they’re not. And I haven’t really noticed any harm reduction centers or anything, just sort of people who are willing to go out there and pass that stuff out. So, yeah, I would definitely say* [the pandemic] *has affected that.*

Other participants commented on the decreased availability of treatment and social services during the pandemic, but did not attribute increased use to these closures. For example, ‘Roland,’ a 55-year-old white man, explained:

[My drug use is the] *worst it’s ever been. I’m 55 years old, I’ve had periods of clean time, scheduled periods of clean time, but the last month, it has progressed to a level that I’ve never had before…. I mean, there’s no place- help is not what it was. I’m not blaming that, I mean, I’m solely responsible for my addiction, no one else is, but* [the pandemic’s] *clearly increased it*.

At the institutional level, harm reduction, treatment, and social services were impacted by pandemic-related temporary closures. For some participants, these service interruptions left them without regular access to harm reduction supplies such as sterile syringes and naloxone despite local shifts toward mobile outreach and telehealth [34]. Participants were not directly asked about access to medications for opioid use disorder (MOUD) during the pandemic, though nearly half of the participants mentioned methadone use. Only one participant (‘Jessika,’ a 33-year-old white woman) noted difficulty accessing MOUD during the pandemic due to financial strain:

[I] *don’t even have enough money to get on the bus to get to the methadone clinic. And that’s really made things really awful. Without getting to the methadone clinic, you have to do something to buy dope.*

Participants noted reasons for reduced access to services that were directly related to the pandemic (e.g., lack of in-person services), indirectly related (e.g., financial strain), and reasons unrelated to the pandemic (e.g., lack of harm reduction supplies at pharmacies). Given this heterogeneity and the focus of participants’ comments on access to institutions rather than underlying policy drivers, reduced access to services has been categorized as an institutional-level factor.

### Public policy level

#### Financial

Several participants indicated that increased financial resources, including Internal Revenue Service (IRS)-issued Economic Impact Payments (‘stimulus checks’), influenced their drug use behaviors. For example, ‘Colton’ shared:

[My drug use has] *definitely gone up. Definitely. Just because so much more free time, and then the stimulus check, too, which, you know, when that came in, I blew through that probably in, like, three days or something like that.*

Though not stated as explicitly relating to IRS-issued Economic Impact Payments, ‘Alisa’ noted that greater financial resources at the beginning of the pandemic contributed to increased use:

*I was using a lot when it* [the pandemic]* first started. I was probably inside more* [and] *I had like a little more money back then to, to use and, and like I said, and being home a lot it was just, you know, there wasn't much to do and nobody was really around.*

#### Job loss and business closures

Several participants indicated the significant impact that job loss during the pandemic had on their drug use patterns. For many participants, this led to an increase in substance use to pass unstructured time. While these factors overlap with individual-level boredom described above, only a subset of participants who described boredom as a driver of increased use overtly linked that boredom to structural factors such as job loss and business closures. For example, ‘Crystal,’ a 50-year old white woman explained:

*Since the pandemic,* [my drug use]* definitely went up triple…just doing a lot more. *[My drug use increased] *‘cause I wasn’t working. I had too much time on my hands. So that’s what I was doing.*

‘Jessika,’ a 30-year-old white woman, similarly tied boredom to job loss:

*Without having work? And being home all the time. Being, like, alone all the time, yeah. I think I’m definitely using more. Because of things that are like, indirectly related to the pandemic.*

Other participants, such as ‘Holly,’ a 30-year-old white woman, noted that business closures unrelated to employment impacted her drug use by causing boredom:

*I do more* [drugs] *now because it’s like, it seems like there’s less to do since the pandemic started. So I do more *[drugs], *yeah. […] Because everything’s closing, yeah.*

Policy-level influences affecting drug use behaviors included financial changes and job loss. While these influences stemmed from policy-level factors, participants linked them to boredom, an individual-level driver of drug use behavior change.

## Discussion

We found that PWUD in Rhode Island experienced changes in their drug use patterns during the pandemic—including increased use, change in drug type, and/or more solitary drug use—that can amplify risk of nonfatal and fatal overdose. Participants identified multiple individual, network, institutional, and policy-level influences of the pandemic on overdose risk behaviors, and our findings suggest that during large-scale crises, such as the COVID-19 pandemic, effective harm reduction strategies should seek to target several levels of influence concurrently.

Our finding that individual-level factors (i.e., depression, anxiety, isolation, and boredom) influenced drug use patterns in ways that could increase overdose risk aligns with previous research on the role of substance use in emotional regulation [35] and with national surveys that documented increases in substance use to cope with pandemic-related stressors [2, 3, 6]. This finding is further corroborated by other qualitative investigations among PWUD during the COVID-19 pandemic [13, 15, 18, 36]. Further, the relationship between boredom and substance use is well-documented, including during the COVID-19 pandemic [14, 15, 37, 38]. Approaches to mitigate feelings of isolation and boredom among PWUD during the pandemic or other large-scale crises could include virtual opportunities for connection among PWUD (e.g., ‘spotting’ or group use via video chat or phone). For example, the BeSafe App, designed by Brave Technology Cooperative in Vancouver, Canada, connects PWUD with a ‘supporter’ whose specific role in supporting harm reduction is determined by the caller [39]. Informal virtual spotting networks have been previously studied [40]; these networks target both individual-level influences of overdose risk behavior (e.g., boredom, isolation) as well as institutional-level influences by disseminating harm reduction resources even during service disruptions [41]. Furthermore, given the influence of depression and anxiety on overdose risk behaviors, current findings indicate a need for more robust integration of mental health services, including low threshold, trauma-informed therapeutic environments in settings that serve PWUD, as well as contingency plans for mental health resources to minimize disruptions to service [42, 43].

Network-level factors were also found to influence overdose risk behaviors in the present study. Participants reported changes in access to their drug of choice, including benzodiazepines and crack cocaine due to drug market changes during the pandemic. Participants reported varying experiences with crack cocaine accessibility during the pandemic, with some indicating increased access and others reporting a decline in availability. In Rhode Island, the number of overdose deaths involving cocaine increased by 48% between 2019 and 2021 [44], suggesting an increase in cocaine present in the local drug supply during this period. Notably, this statistic does not differentiate between overdoses involving cocaine only, or those in combination with other drugs (for example, fentanyl and cocaine)—this highlights the need for better real-time data on local drug supply in Rhode Island. While access to crack cocaine is influenced by international and state-level drug market dynamics, it is likely that other factors play a role, including community-level availability, seller behavior, and individual characteristics.

Participants indicated that fentanyl was increasingly present in the drug supply during the pandemic. This is consistent with other studies, which have documented shifts in local drug markets during the pandemic including increased fentanyl availability, fentanyl contamination/reduced drug purity, and reduced access to preferred substances among PWUD, both across the USA [12, 13, 19, 27, 36, 45] and internationally [11, 14, 15, 38, 46]. The continued proliferation of fentanyl in illicit drug markets underscores the need for states to establish sustainable funding to expand community-based distribution of naloxone [47, 48] and fentanyl test strip distribution [49–51], as well as the need to establish overdose prevention sites [52, 53].

Additionally, at the network level, several participants reported that social network changes caused by housing disruptions precipitated increases in their overdose risk behaviors during the pandemic. A similar relationship among housing, social networks, and substance use was also described in a pre-pandemic cohort of adults in unstable housing in Vancouver, BC [54], and two prospective cohort studies in the same city identified unstable housing as a significant predictor of all-cause mortality among people who inject drugs [55]. Policies to ensure access to stable housing among PWUD, particularly during destabilizing crises such as the COVID-19 pandemic, must be prioritized, including rent stabilization [56], eviction moratoria [57], rent/mortgage deferrals [58], and sustainable temporary housing [59].

At the institutional level, several participants described difficulty accessing harm reduction street outreach efforts during treatment and harm reduction center closures. Service disruptions were also noted in other studies among PWUD during the pandemic [12–14, 45]. These service disruptions, while occurring at the institutional level, were clearly affected by pandemic closure policies, illustrating the interactive nature of the institutional and policy levels. To ensure that harm reduction and treatment services are not disrupted during pandemics and other disasters, organizations providing these services should be appropriately funded, be considered essential, and encouraged to adopt flexible operations. These operational changes may include shifting toward outreach/mobile services, telemedicine expansion, and mail delivery of harm reduction equipment [60]. In Rhode Island, while several harm reduction organizations remained open for in-person services in the early pandemic, organizations also shifted toward mobile street outreach, naloxone and fentanyl test strip delivery, reduced hours of operation, and virtual recovery meetings [9, 34].

On the public policy level, participants cited a lack of employment and business closures as influencing their overdose risk behaviors. Many participants linked these structural factors to having ‘too much time’ or ‘nothing to do’ (i.e., boredom on the individual level), highlighting the inherent interactivity of levels across the social-ecological model. Some participants noted that short-term increases in financial resources (including the receipt of Economic Impact Payments) influenced their drug use. Previous research has documented increases in substance use following cash payments [61, 62], including an increase of overdose deaths following EIP distribution in Ohio [63]. In Rhode Island, a previous study did not find an association between the proportion of residents at the neighborhood-level receiving monthly income assistance and overdose mortality in the week following check receipt, though elevated overdose mortality in the first week of the month was associated with the proportion of residents living in unaffordable housing [64]. To mitigate any potential drug-related harms surrounding cash payments, harm reduction outreach should be intensified in times surrounding synchronized cash assistance disbursal [65], such as pandemic-era Economic Impact Payments in the USA. Additionally, policies that alleviate housing-related stressors and increase housing stability, such as those described above, could further mitigate potential payment-coincident, drug-related harms.

Importantly, all participants in the present study named factors corresponding to *more than one* social-ecological level when discussing changes in drug use patterns associated with increased overdose risk. This suggests that interventions to reduce overdose risk behaviors during the ongoing pandemic and future public health crises would be more effective if they engage several levels of social organization at once (i.e., ‘multi-level interventions’). For example, virtual periodic check-ins from trained care coordinators or peers could identify housing needs, harm reduction (including ‘spotting’) needs, combat isolation and boredom, and connect PWUD with resources to mitigate drug- and housing-related harms. Such interventions would address individual, social network, institutional, and institutional influences on overdose risk behavior. Multi-level interventions such as these will require coordination among PWUD, community members, harm reduction and treatment organizations, and local policymakers.

Our finding that the COVID-19 pandemic influenced drug use behavior among PWUD aligns with other qualitative investigations during the pandemic [11–19], and with previous research on the impact of ‘big events’ (i.e., natural disasters, financial crises) on substance use patterns [66]. By framing these influences through a social-ecological model, our findings highlight the interactive and mutually reinforcing nature of the levels of influence on drug use behaviors. Examining the impact of the pandemic on PWUD through this novel lens, the findings presented here underscore and reinforce the importance of multi-level interventions among PWUD during global crises.

## Limitations

Several limitations should be considered when interpreting these findings. First, the sociodemographic characteristics of this sample reflect those of the state of Rhode Island (i.e., majority non-Hispanic white, cisgender, and heterosexual). Hence, these results may not represent the unique experiences of communities of PWUD with greater diversity in race/ethnicity, gender identity, and sexuality during the pandemic. Notably, most qualitative studies with PWUD during the COVID-19 pandemic have enrolled majority-white samples, and few report gender and sexuality of participants, highlighting the need to replicate research examining the impacts of COVID-19 on drug use practices in more diverse samples. Second, participant recall and/or social desirability bias may have distorted self-reported risk behaviors during the pandemic. Nonetheless, self-reported drug behaviors are generally considered valid for a range of substances (67–69).

## Conclusions

In a sample of PWUD in Rhode Island, the present study identified increased overdose risk behaviors during the ongoing COVID-19 pandemic. Participants identified multiple factors influencing overdose risk behaviors at the individual, network, institutional, and public policy level. While the methods employed here preclude causal inference, our findings suggest key levers for intervention to improve safety among PWUD during local and global crises. In this sample, the most reported reasons for increased overdose risk behaviors were: boredom (individual level), changes in the local drug supply (network level), reduced access to services (institutional level), and job loss and/or business closures (public policy level). Further, our findings suggest the COVID-19 pandemic impacts overdose risk behaviors at several levels of influence at once, indicating that interventions to reduce overdose risk behaviors may be more effective if they engage several levels of influence concurrently.

## Data Availability

The datasets that were used and analyzed as part of the present study are available from the corresponding author on reasonable request.

## Abbreviations

COVID-19: Coronavirus disease 2019
PWUD: People who use drugs
RAPIDS: Rhode Island Prescription and Illicit Drug Study
MOUD: Medications for opioid use disorder
